# Long-Acting Risperidone Dual Control System: Preparation, Characterization and Evaluation In Vitro and In Vivo

**DOI:** 10.3390/pharmaceutics13081210

**Published:** 2021-08-05

**Authors:** Xieguo Yan, Shiqiang Wang, Kaoxiang Sun

**Affiliations:** 1 School of Pharmacy, Collaborative Innovation Center of Advanced Drug Delivery System and Biotech Drugs in Universities of Shandong, Key Laboratory of Molecular Pharmacology and Drug Evaluation (Yantai University), Ministry of Education, Yantai University, Yantai 264005, China; 2 Shenzhen Sciencare Medical Industries Co., Ltd., Shenzhen 518118, China; wangshiqiang@sciencare.cn

**Keywords:** risperidone, microsphere, tablet, implant, sustained-release, stepwise regression

## Abstract

Schizophrenia, a psychiatric disorder, requires long-term treatment; however, large fluctuations in blood drug concentration increase the risk of adverse reactions. We prepared a long-term risperidone (RIS) implantation system that can stabilize RIS release and established in-vitro and in-vivo evaluation systems. Cumulative release, drug loading, and entrapment efficiency were used as evaluation indicators to evaluate the effects of different pore formers, polymer ratios, porogen concentrations, and oil–water ratios on a RIS implant (RIS-IM). We also built a mathematical model to identify the optimized formulation by stepwise regression. We also assessed the crystalline changes, residual solvents, solubility and stability after sterilization, in-vivo polymer degradation, pharmacokinetics, and tissue inflammation in the case of the optimized formulation. The surface of the optimized RIS microspheres was small and hollow with 134.4 ± 3.5 µm particle size, 1.60 SPAN, 46.7% ± 2.3% implant drug loading, and 93.4% entrapment efficiency. The in-vitro dissolution behavior of RIS-IM had zero-order kinetics and stable blood concentration; no lag time was released for over three months. Furthermore, the RIS-IM was not only non-irritating to tissues but also had good biocompatibility and product stability. Long-acting RIS-IMs with microspheres and film coatings can provide a new avenue for treating schizophrenia.

## 1. Introduction

Schizophrenia is a chronic and severe mental illness that affects more than 20 million people worldwide [1]. Clinically, it often manifests as syndromes with different symptoms, involving various obstacles in perception, thinking, emotion, behavior, and coordinating mental activities. The course of the disease is usually long with repeated episodes, exacerbations, or deteriorations, and some patients eventually develop degeneration and mental disability [2]. Patients with schizophrenia are 2–3 times more likely to die at an early age than the general population [3]. Long-term medication affects the quality of life of patients, affects family relationships, and leads to drug compliance [4]. The pathogenesis of schizophrenia has yet to be fully characterized; however, previous studies have reported that certain genes [5] or environmental factors [6] affect the onset of schizophrenia [7,8].

Second-generation antipsychotic drugs, also known as atypical antipsychotics, have fewer side effects than typical first-generation antipsychotics [9]. Risperidone (RIS) is a derivative of benzisoxazole, which is a combination of the chemical structures of haloperidol and riserin; it can selectively antagonize 5-HT2A receptors and dopamine D2 receptors in the midbrain and limbic system. RIS has a promising effect on both positive and negative symptoms of schizophrenia. The extrapyramidal reactions (EPSs) of RIS are lighter and have lower resistance. RIS also has better receptivity and considerable advantages over other atypical antipsychotics [10]. The primary metabolite of RIS is 9-hydroxy-RIS (paliperidone), which has a pharmacological effect equivalent to that of RIS in the body [11,12].

RIS has low solubility and high permeability in water and belongs to category II of the Biopharmaceutical Classification System [13]. The dissolution rate of RIS is a limiting factor for its absorption; however, it also has a first-pass effect and a bioavailability of 70%. Moreover, since the antipsychotic EPS is related to blood drug concentrations [14], a reduction in the dose of the drug without affecting the efficacy can reduce the incidence of EPS. A long-acting RIS injection for atypical schizophrenia was introduced in 2002, and it is now the only long-term injection that can be used for both schizoaffective disorder and bipolar disorder [15]. The RIS long-acting injection dosage is only 38.1 mg/g, with inactive substances comprising copolymers of polylactide and hydroxyacetic acid [16]. The formulation has a delayed release (cumulative release < 1%) within 2–3 weeks of administration and requires the administration of oral RIS tablets. Moreover, steady blood concentrations were achieved within 6–8 weeks after four doses, with no significant advantage over the dosage of oral tablets (2–4 mg/day) for long-acting injections (25–50 mg/week). Furthermore, polyurethane and poly lactic-co-glycolic acid (PLGA) have been used as slow-release materials. Researchers have recently developed long-acting RIS microspheres, which gradually release RIS for up to 3 months and do not have the drawbacks of oral tablets [17,18]. Although these studies did not determine the ideal long-acting preparation of RIS, they provided guidance for further studies.

Polylactic acid (PLA) has two optical forms, namely d-lactide and l-lactide. It is a biodegradable hydrophobic aliphatic polyester widely used in biomedical fields [19]. The physical properties and biodegradability of PLA can be changed by modulating hydroxy acid copolymers or racemate the d-and l-isomers, with degradation rates depending on crystallinity [20]. PLA has a longer degradation time and better stability than PLGA and is often used to produce ultra-long-lived products. PLA is widely used in sutures [21], bone implants [22], screws [22], and continuous drug delivery [23,24,25]. LUPRON DEPOT^®^ (4/6 months) [26], which uses PLA as a slow-release material, has been approved by the U.S. Food and Drug Administration for various diseases, including prostate cancer, endometriosis, myoma, and precocious puberty. Several preclinical studies have confirmed the safety, biodistribution, and efficacy of biocompatible implants in animal models [27,28]. These biodegradable excipients are more suitable for producing biocompatible long-acting preparations, but occasional adverse reactions may occur [29,30], and the dosage of the additives should be reduced as much as possible.

Mathematical models simplify abstract complex practical problems into a reasonable mathematical structure. They can explain certain objective phenomena, predict the law of future development, and provide strategies for controlling the results of a specific phenomenon. Their remarkable advantages ensure that they are widely used in various fields of drug research, including drug research and development [31], stability prediction [32], pharmacokinetics [33], and pharmacoeconomics [34].

In this study, we sought to prepare an innovative long-acting implant to reduce the dosage and improve the bioavailability of RIS. We employed the microsphere compression process to avoid oral supplementation of RIS and construct a RIS-IM capable of releasing the drug with zero-order kinetics in the body for more than three months, for high drug loading, and as an ideal formulation with high entrapment efficiency. Cumulative release, drug loading, and entrapment efficiency were used as evaluation indicators to evaluate the effects of different pore formers, polymer ratios, porogen concentrations, and oil–water ratios on the RIS-IM. In addition, we built a mathematical model to identify an optimized formulation by stepwise regression. We used X-ray diffraction (XRD) to assess the crystals formed, differential scanning calorimetry (DSC) to analyze the thermal stability, gas chromatography (GC) to determine the solvent residue of the preparation, and a desktop electron microscope to observe morphology. Sprague–Dawley rats were used to evaluate the pharmacokinetics of the optimized formulation, to conduct an issue irritation test, and to determine the stability.

## 2. Materials and Methods

### 2.1. Materials

RIS was purchased from Jiangsu Nhwa Pharmaceutical Co., Ltd. (Jiangsu, China). PLA (Mw 50,000, inherent viscosity 0.45–0.55 dL/g) was gifted by ScienCare Medicine Co., Ltd. (Shenzhen, China). PVA (88% hydrolysis, degree of polymerization 24) was purchased from Jiangxi Alfa Hi-Tech Pharmaceutical Co., Ltd. (Pingxiang, Jiangxi, China). Magnesium stearate was purchased from Anhui Sunhere Pharmaceutical Excipients Co., Ltd. (Anhui, China). Dichloromethane and ethyl acetate were purchased from Nanjing Chemical Reagent Co., Ltd. (Nanjing, China). Acetonitrile and methanol were purchased from Merck (Darmstadt, Germany). All other chemical reagents were of analytical grade.

Sprague–Dawley rats were purchased from Beijing HFK Bioscience Co., Ltd. (Beijing, China). All the animal experiment protocols were approved by the Ethical Committee of Animal Experimentation of the Shenzhen People’s Hospital (Shenzhen, China) and are in compliance with EC Directive 2010/63/EU and the NIH guidelines on animal welfare.

### 2.2. Preparation of Microspheres

A mixture of RIS and PLA was added to the dichloromethane (DCM) solution and dissolved by stirring using a magnetic stirrer (RCT Basic Package; IKA, Staufen, Germany). After the pore former (sodium chloride or sucrose) was dissolved in distilled water, the dichloromethane solution with RIS and PLA mixture was added to it, vortexed using a vortex mix (Hula Dancer; IKA, Staufen, Germany) for 30 s, and the primary emulsion was obtained by ultrasound (Branson550; Emerson, Saint Louis, MO, USA) for 20 s. A mixture of 0.5% polyvinyl alcohol and 5% sodium chloride was used as the external phase solution. The primary emulsion was stored at 4–8 °C for 1 h with continuous stirring using the magnetic stirrer and then injected into the external phase solution using a disposable syringe and injection pump (PHD ULTRA; Harvard Apparatus, Holliston, MA, USA). The microspheres were filtered and collected after 24 h. The microspheres were vacuum-dried, sieved (China Standard Sieve Series, Shaoxing, Zhejiang, China), and stored in a dryer. Table 1 summarizes the formulation and dosage ranges of the different batches of microspheres.

### 2.3. Implant Preparation

The microspheres were mixed with 0.5% magnesium stearate (*w*/*w*, MgSt) using a hopper mixer (KCLD-3; Beijing Kaichuangtonghe Technology Development Co., Ltd., Beijing, China) for 4 min, and was compressed to form a tablet. The 3-mm punches of heads were assembled using a tablet press machine (ZP-10A; Beijing Gylongli Sci and Tech, Beijing, China). After tablet compression, the tablets were coated with 2% ethyl acetate solution of PLA (BGW-C; Zhejiang Xiaolun Pharmaceutical Machinery, Wenzhou, Zhejiang, China) and dried for 1 h, followed by vacuum drying.

### 2.4. Mathematical Model

The correlation coefficient was determined by linear regression analysis of in-vitro cumulative release and time using the least square method. By taking the correlation coefficient as the response value, according to the difference between the AIC criterion [35] and regression coefficient, the best model was established by stepwise regression, and the best formula was predicted on the basis of the model.

### 2.5. Surface Morphology Analysis

The surface of microspheres was observed using a tabletop electron microscope (TM 4000; Hitachi, Tokyo, Japan) by backscattering electron signals. The sample was laid flat on conductive double-sided tape. Under negative pressure, the sample was then observed at a slow speed at an excitation voltage of 15 kV using Chg-up Red(L) mode.

### 2.6. Particle Size and Distribution

According to the principle of dynamic light scattering, the particle size and polydispersity index were measured using laser-diffraction-size analyzers (Bettersizer 2600; Bettersize Instruments, Liaoning, China). Product samples (3 g) were placed on the tray and the oscillation frequency was 5, the tray height was 4 mm, and the air pressure was 0.5. All models were repeated three times (n = 3), and the vacuum cleaner collected the samples after each cleaning.

### 2.7. Drug Loading and Entrapment Efficiency

A total of 220 mg of the powder was accurately weighed and placed in a 100-mL measuring flask. Twenty milliliters of 0.1 mol/L hydrochloric acid solution was added for ultrasonication for 30 min to dissolve the powder. After cooling to room temperature, 0.1 mol/L hydrochloric acid solution was used to dilute the sample to the mark. The sample was shaken well and filtered. Subsequently, 1.0 mL of the subsequent filtrate was placed in a 10-mL measuring flask, 0.1 mol/L hydrochloric acid solution was added to the mark, and the flask was shaken. The microspheres were washed with anhydrous ethanol to remove excess RIS before measurement.

Octadecylsilane-bonded silica gel was used as a filler (Symmetry^®^ C18, Waters, MA, USA) and 1.1 g/L sodium octane sulfonate solution (adjusted pH to 2.3, with phosphoric acid)—acetonitrile (70:30) was used as the mobile phase. The column temperature was 40 °C, the flow rate was 0.8 mL/min, the injection volume was 20 µL, and the detection wavelength was 275 nm. The free risperidone and risperidone microspheres were filtered with sand cores and washed twice with 25% ethanol solution (weight ratio, 1:10). The drug loading and entrapment efficiency were calculated as follows [36]:

drug loading% = (weight of drug entrapped/weight of sample used) × 100%

entrapment efficiency% = (experimental drug loading/theoretically drug) × 100%

### 2.8. Thermal Analyses

The melting point was determined by DSC (DSC4000; PerkinElmer Ltd., Shelton, CT, USA), including the RIS, PLA, MgSt, a mixture of RIS/PLA/MgSt, and RIS-IMs. The sample (5 mg) was weighed in a standard aluminum pan, covered, and kept at 40 °C for 1 min. The sample was then heated from 40 to 200 °C at 10 °C/min and cooled to room temperature. Every sample was measured twice. The first measurement was used to offset the thermal memory of the instrument, and the output of the second measurement was used. The apparatus was calibrated using 99.99% indium.

### 2.9. X-ray Powder Diffraction

XRD was used to analyze the physical states of the samples. We used Cu-Ka rays of XRD (Empyrean, Malvern Panalytical Ltd., Worcestershire, UK), voltage 40 kV, current 40 mA, divergence slit 1/8°, anti-scattering slit 1/4°, and 7.5 mm to obtain the XRD pattern of RIS, the mixture PLA and MgSt, RIS-IMs, respectively. Samples (1 g) were crushed using a mortar and pestle and added to the sample holder. The diffraction patterns were scanned at a rate of 0.03°/min in the range of 2–60°.

### 2.10. Residual Solvent

The residual dichloromethane in the formulation was analyzed by headspace GC (GC2014; Shimadzu, Tokyo, Japan). GC was performed using a capillary column with 6% cyanopropyl phenyl, 94% dimethyl polysiloxane, and a flame ionization detector. After grinding, the product was accurately weighed (500 mg), and placed in a top empty bottle. Isopropanol internal standard solution (5 mL) was added to the bottle, which was kept at 40 °C in a water bath to dissolve the product. The carrier velocity was 1.0 mL/min, and the shunt ratio was 1:5. The injection temperature was 180 °C, the detector temperature was 250 °C, and the starting temperature was 45 °C. The temperature was increased to 180 °C at a rate of 5 °C/min. At the same time, the sample bottles were heated to 85 °C for 30 min.

### 2.11. In-Vitro Experiments

The release medium contained 80 mL of phosphate buffer solution (pH 7.4). After sterilization by Co60 irradiation, the samples of the optimized formulation implants were kept in a constant temperature shaker (ZWF-110 × 50; Labwit Scientific Pty., Ltd., Shanghai, China). The shaker was set at 37 ± 0.5 °C and the oscillation frequency was 50 rpm. Samples were taken on the first day and then every three days from the third day, and the medium was changed every time.

### 2.12. In-Vivo Experiments

#### 2.12.1. Grouping and Administration

Forty-two Sprague–Dawley rats weighing 180 ± 20 g and aged 6–8 weeks were randomly assigned to cages divided into male and female halves. The rats were administered 2% pentobarbital sodium solution at 40 mg/kg weight before surgery. After the rats were anesthetized, surgical scissors were used to cut an opening of about 2 cm on the back of the rat, and RIS-IMs were kept in it. A 3M bio-adhesive was added to the wound and closed with pressure for 30 s, followed by rubbing with 75% ethanol. The first group of rats (n = 30) was administered doses equivalent to 50 mg RIS [18], of which 12 rats were used only for pharmacokinetics and 18 were used for degradation studies in polymers. The second group of rats (n = 12) received a dose equivalent to 25 mg RIS.

The blood samples were collected from the jugular vein of the rats (n = 12) at the planned time. The collected blood samples were centrifuged for 5 min at 7000 rpm, and the plasma was collected and stored at −20 °C.

#### 2.12.2. Biological Sample Analysis

The plasma sample (50 μL) was transferred to a centrifuge tube, to which 200 μL buspirone methanol solution (30 ng/mL) was added. The tube was vortexed for 3 min and centrifuged at 1500× *g* for 10 min, and the supernatant was analyzed by liquid chromatography-tandem mass spectrometry (LC-MS/MS) (6470; Agilent, Santa Clara, CA, USA).

The LC-MS/MS system included an Agilent 1296 high-performance liquid chromatography system (HPLC 1290; Agilent, Waldbronn, Germany) and an electrospray ionization (ESI) ion source. Chromatographic separation was performed using the Agilent RRHD Eclipse Plus C18 column (3.0 × 50 mm, 1.8 μm). The mobile phase was 0.1% formic acid aqueous solution-acetonitrile (65:35, *v*/*v*), the flow rate was 0.3 mL/min, and the injection volume was 0.8 μL. The MS conditions were as follows: ESI, capillary voltage 4000 V, drying gas temperature 300 °C, drying gas flow rate 5 L/min, sheath gas temperature 350 °C, sheath gas flow rate 12 L/min, and nebulizer pressure 35 psi. Using MRM mode, the ion pairs were scanned at *m*/*z* 411.2 → 190.9 (risperidone), *m*/*z* 427.2 → 206.9 (9-OH- risperidone), and *m*/*z* 386.1 → 150.1 (internal standard, Buspirone) under a positive ion.

The calibration curves were established for each analysis, and the correlation coefficient was R^2^ > 0.99. The lower limit of RIS was 0.48 ng/mL, and the lower limit of 9-hydroxy- RIS was 0.54. The mean recoveries of RIS and 9-hydroxy- RIS in plasma samples from low to high concentrations were higher than 93.1% and 93.6%, respectively, and the intraday and diurnal accuracy coefficients were less than 15%.

#### 2.12.3. Statistical Analysis

PKSolver (V2.0, China Pharmaceutical University, Nanjing, Jiangsu, China) was used to process the blood drug concentration data (risperidone and 9-OH- risperidone) at various time points. Selecting the non-compartmental model, we calculated the pharmacokinetic parameters t_1/2_, T_max_, C_max_, AUC_0-t_, AUC_INF_, MRT_Last_, Vz, Cl, and the drug concentration-time curve [37].

### 2.13. Polymer Degradation Test

The molecular weight and polydispersity of the polymers were determined after removing implants from the bodies of the first group of rats (n = 3). The implants were accurately weighed, dissolved in tetrahydrofuran, and made into a 1.5 mg/mL solution. Using a C4 column (Mono GPC-500, Sepax, Newark, DE, USA) with tetrahydrofuran as the flow phase, the flow velocity of 0.5 mL/min, and the differential refractive index detector, 20 μL of the solution was injected into the HPLC system (SCL-20Avp; Shimadzu, Tokyo, Japan) for determination [38]. Recording chromatograms were calculated using GPC software. The sampling times were 0, 30, 45, 60, 90, and 120 days.

### 2.14. Irritation Evaluation

The rats (n = 3) were euthanized for the polymer degradation test every time. We took the muscle of one of the rats for pharmacological tissue sectioning. Tissue specimens of the implant contact site sized 0.5 × 3 cm were cut out. The tissue samples were fixed with 10% formalin, dehydrated with an ethanol gradient, embedded in paraffin, and stained with hematoxylin and eosin. All the samples were examined using an optical microscope (BX43, Olympus, Tokyo, Japan), and pathological changes were evaluated [39]. The tissue sections were observed at 0, 15, 30, 60, 90, and 120 days.

### 2.15. Stability of Products

We studied the experiment of affecting factors and accelerated stability of 6 months (40 °C × 65% RH) according to the International Council for Harmonisation of Technical Requirements for Pharmaceuticals for Human Use (ICH). The content and change of the related substances were evaluated, and the test conditions were adopted using the drug loading method [40].

## 3. Results and Discussion

### 3.1. Microsphere Preparation

**Figure 1:**
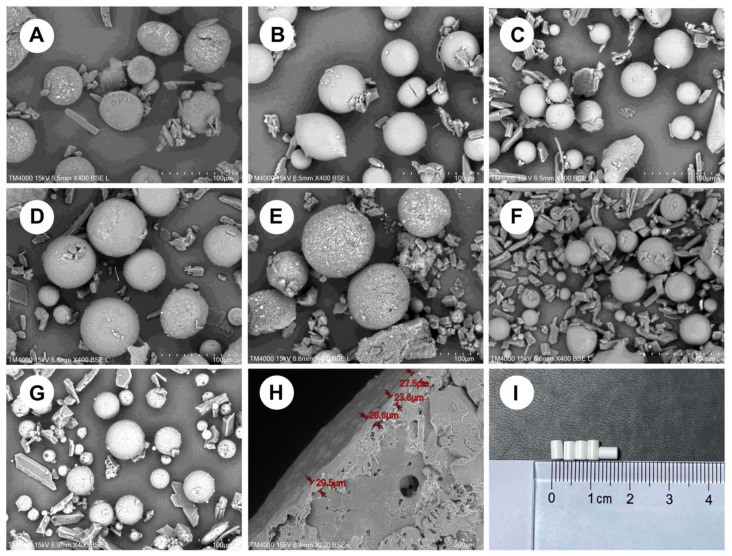
Electron micrographs of microspheres prepared with different formulations: (**A**) F1; (**B**) F2; (**C**) F3; (**D**) F4; (**E**) F5; (**F**) F6; (**G**) the optimized formulation; (**H**) the transverse section of the implant of the optimized formulation, and (**I**) the appearance of the optimal formulation implant.

**Figure 2:**
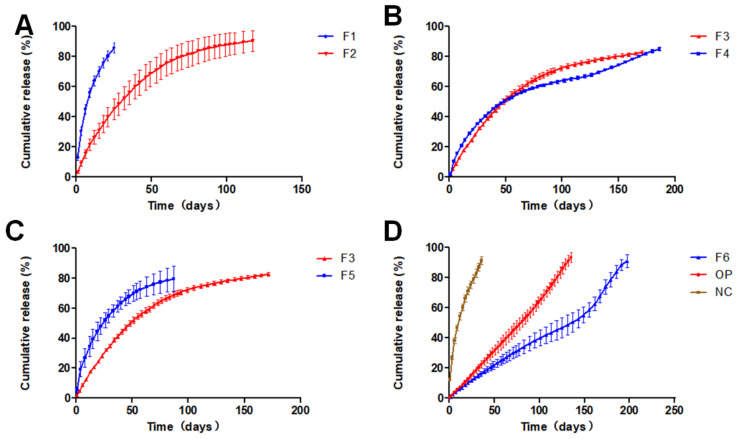
(**A**) Comparison of the dissolution of different pore formers on implants. (**B**) The effect of different risperidone/PLA ratios on the dissolution of implants. (**C**) The effect of different porogen concentrations on the dissolution of implants. (**D**) F6 was prepared using the single emulsion method; OP is the optimized formulation implant; NC is an uncoated optimized formulation tablet.

As shown in Figure 1a,b, the microspheres were made of two pore formers with similar size and surface morphology. At the same concentration, the surface of F1 microspheres prepared using sodium chloride as a pore former had a uniform distribution of holes, whereas the surface of F2 microspheres prepared using sucrose as a pore former was relatively smooth. According to the Noyes–Whitney equation: dC/dt = KS (C_S_ − C), where dC/dt is the dissolution rate, K is the dissolution rate constant, S is the drug surface area, Cs is the drug dissolution, and C is the drug concentration in the solution. When the dissolution rate is constant, an increase in the specific surface area increases the dissolution rate. As illustrated in Figure 2a, this process causes the F1 implants to dissolve faster than the F2 implants. The solubility of sucrose is remarkably higher than that of sodium chloride; therefore, sucrose was used as the internal water phase. Controlling the dissolution rate of the implant was challenging. Therefore, sodium chloride was selected for further studies.

Figure 2b indicates that RIS/PLA (*w*/*w*) was in the range of 2:3–1:1, and the release time of two implant formulations was equal, but the release procedures were different. The F3 implant had a smooth release curve, but the F4 implant had a reverse S release curve. Figure 1c,d microspheres had similar surfaces. As the microspheres were compressed and PLA swells in water, squeezing occurs inside the implant to reduce the gap. The dissolution of RIS in the implant was blocked, and the time was prolonged, especially at the high PLA percentage. As PLA was degraded, the release rate increased.

Compared with F3, the drug loading of the F5 implant had a more significant gap with the drug loading of the microspheres, indicating that the amount of drug outside the microspheres was more. The high concentration of sodium chloride promoted the increase in the number of pores and pore size of the microspheres. In these conditions, RIS was transferred from the inside to the outside, resulting in a faster release.

F6 was prepared using a single emulsion without a porogen. Figure 2d shows that the release period of the formulation was substantially increased. The early release of the F6 implant followed level 0, and the release accelerated after 150 days. The result was because of the increase in the internal pore size caused by PLA degradation.

The correlation between the release curves of formulations was calculated by the least square method. The correlation coefficient was an evaluation index that indicated the effect of release behavior of various factors on the implants. Regression modeling was performed using five independent variables, namely the risperidone/PLA weight ratio, DCM volume, PLA concentration, sodium chloride concession, and *w*/*o* volume ratio of pore-forming agents. The correlation of the dissolution curves was taken as the dependent variable. R language (R 3.6.2) function drop1 [41] and manual elimination were used to build the adjusted optimized model R^2^ = 0.977–1.311 × *w*/*o*. The results (Table 2) suggest that the *w*/*o* ratio significantly affects the correlation coefficient and has no relationship with other independent variables. A unit reduction in the *w*/*o* ratio could increase R^2^ by 1.311. Table 3 shows that the global statistics of the model diagnosis and the *p*-values of other tests were greater than 0.05, and the data meets all the assumptions of linear regression. The optimized implant formulation was as follows: 7.5 g of RIS, 7.5 g of PLA, 25 mL of DCM, 5% sodium chloride as a pore former and a 1:25 *w*/*o* ratio. The results (Table 4 and Figure 2d) show that the correlation coefficient of the implant prepared by the optimized formulation was R^2^ = 0.9967, and the release time was more than 130 days In Vitro.

The influence of the coating layer of the optimized formula was significant (Figure 2d). Although the uncoated optimized formulation tablet without coating has the same structure as the optimized formulation implant, its release time was less than 50 days. The formulation was performed by a dual control system, and the risperidone was subject to the double restrictions of the sustained release matrix and film control. The solution entered the inside of the implant through the coating film and dissolved the free risperidone. The risperidone present in the microspheres subsequently dissolved out of the microspheres to replenish the lost risperidone. The space between the matrix microspheres and the coating film became a buffer zone. In case the microspheres were burst, there was still a buffer area, and the release of the implant was stable. The dissolution of the uncoated optimized formulation tablet and the optimized formulation implant in Figure 1 was an example.

### 3.2. Thermal Analysis

**Figure 3:**
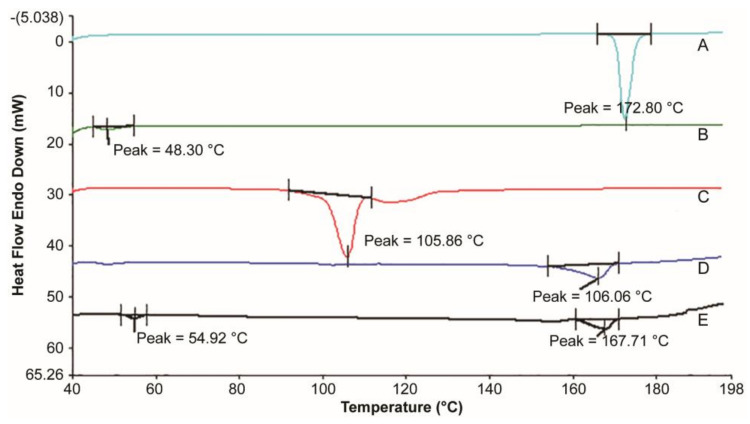
Thermal analysis diagram between risperidone and excipients. Thermal analysis curve. (**A**) Risperidone. (**B**) Polylactic acid. (**C**) Magnesium stearate. (**D**) The physical mixture of risperidone/polylactic acid/magnesium stearate. (**E**) Risperidone implant.

**Figure 4:**
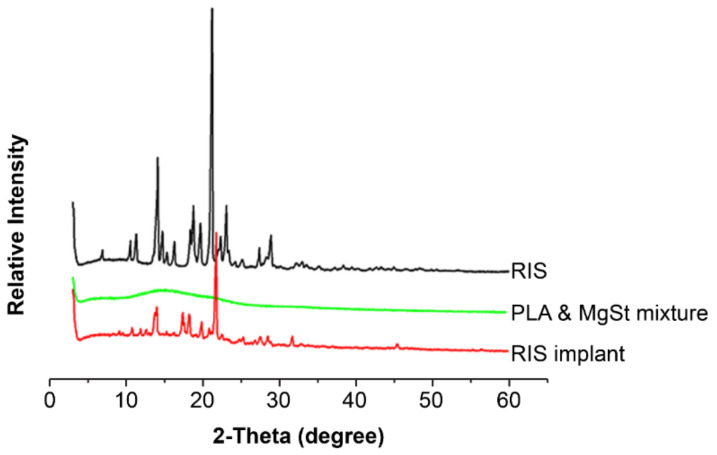
X-ray diffraction pattern between risperidone and excipients. RIS, risperidone; PLA, polylactic acid; MgSt, magnesium stearate.

Figure 3 shows that the RIS had a single melting peak at 172.8 °C in the range of 40–200 °C, consistent with the literature [42]. The glass transition temperature (Tg) of PLA is 48.30 °C. The melting point of RIS-IM reduced from 172.80 to 167.71 °C, and the Tg of the polymer increased from 48.30 to 54.92 °C because of the production of coprecipitate. The physical mixture of RIS/PLA/MgSt reduced from 172.80 to 166.06 °C, since the solid solution was formed during the heating process. Regardless of whether it is a solid solution or a coprecipitate, it is a type of solid dispersion. RIS molecules were dispersed in PLA molecules, and the crystal structure had not changed [43,44]. The XRD pattern of RIS-IM (Figure 4) confirmed that the crystal form of the RIS preparation process did not change, and the process did not affect the stability of RIS.

### 3.3. Residual Solvent

Dichloromethane is an oil-phase solvent used in the preparation of microspheres, and ethyl acetate is the solvent used for coating. According to ICH, dichloromethane is a second-class solvent with a limit of 0.06%, while ethyl acetate is a third-class solvent with a limit of 0.5%. The residual solvent content of the implant was analyzed by GC. The results showed that dichloromethane and ethyl acetate contents were 0.015% and 0.299%, respectively, all of which were less than the permissible limit.

### 3.4. In-Vitro Dissolution

**Figure 5:**
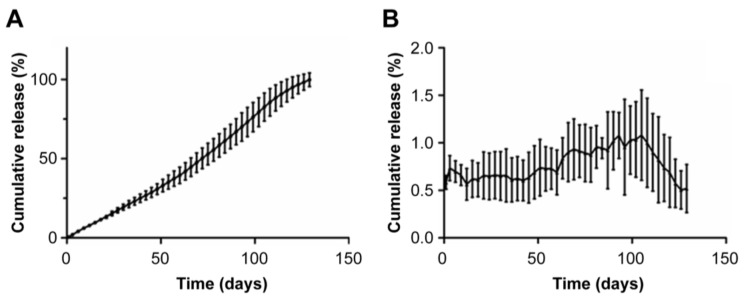
Dissolution of the optimized formula after irradiation sterilization In Vitro. (**A**) Cumulative release curve of the risperidone implant In Vitro. (**B**) Daily dissolution curve of the risperidone implant In Vitro.

The implant was used In Vivo and requires sterility assurance. Irradiation is an effective sterilization method, but it will produce a certain amount of heat to affect PLA. It is necessary to investigate the release behavior of the preparation after irradiation sterilization. The optimized formulation implants were irradiated with Co60. We performed linear regression with cumulative release and time and used EXCEL to get the model with cumulative release% = 0.797 × Time − 4.369 and the correlation coefficient R^2^ = 0.990. The result (Figure 5) indicated that RIS-IM was released In Vitro with zero-order kinetics. The average daily release rate of RIS-IM was stable and between 0.6% and 1.8%, and the initial average daily release rate was 1.0%. In the early stage of dissolution, PLA was less affected by the medium, and the average daily dissolution rate was stable. After 20 days, the PLA on the surface of the implant was eroded, the diffusion distance of RIS was reduced, and the average daily dissolution rate was increased, peaking at approximately 80 days. Subsequently, the rate of change in the amount of residual drug in the implant was less than the erosion rate of PLA, and the average daily release rate decreased until it was ultimately released.

### 3.5. In-Vivo Pharmacokinetics

**Figure 6:**
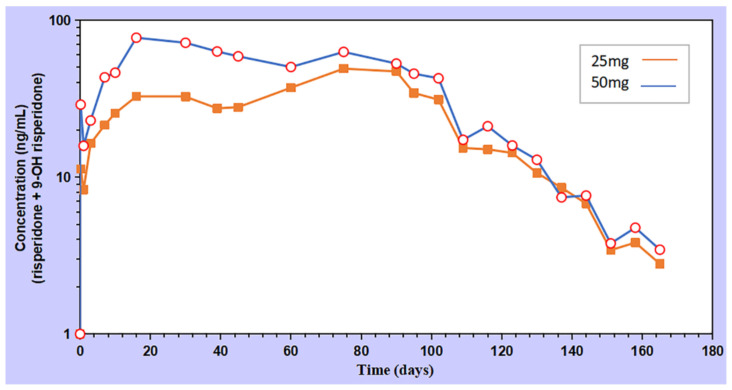
The pharmacokinetic curve of the implants.

The blood concentration of the 50 mg RIS-IM increased on the first day, peaked (Cmax) on day 16, and then decreased. After 75 days, the blood concentration appeared as the second peak, gradually reduced, and almost disappeared after 164 days. The drug release behavior of 25 mg RIS-IM In Vivo was similar to that of 50 mg RIS-IM, except for the Cmax that was delayed to the second peak time. The two doses of RIS-IM exhibited a sustained-release effect, and the result is shown in Figure 6.

There was no release lag phenomenon in the early stage of administration, meaning that oral supplementation was no longer required; moreover, RIS can be released smoothly for more than three months or even reach a six-month release period. The PKSolver software processed the data, and pharmacokinetic parameters (Table 5) showed that the implants with RIS dosages of 25 and 50 mg had C_max_ values of 49.07 and 77.22 ng/mL and an AUC_0_-t of 419,720 and 6593.87 ng/mL·d, respectively.

### 3.6. Polymer Degradation Analysis

**Figure 7:**
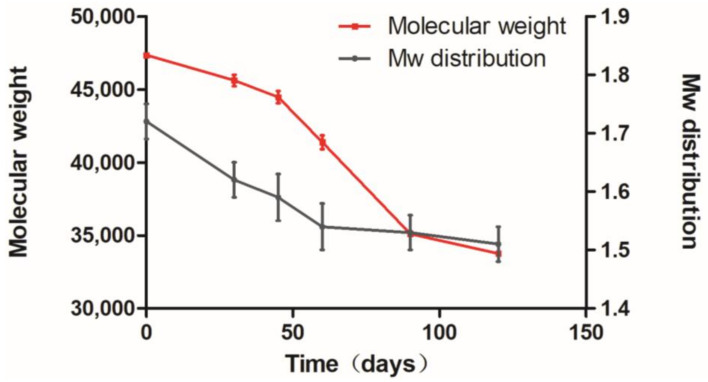
Degradation of polylactic acid in implants In Vivo.

The RIS-IMs (n = 3) were taken regularly from the body to detect PLA degradation. According to the results (Figure 7), the curve was divided into three stages: 0–40, 40–90, and beyond 90 days. The degradation rate in the second stage was faster than that in the early stage, while it slowed down in the third stage. The mean molecular weight of the implant was about 35,000 after 120 days. In the first stage, the unstable bonds of the main chain of PLA were hydrolyzed, causing an increase in the number of hydroxyl groups and a slight decrease in the molecular weight. The second stage reduced the number of carbonyl groups of PLA and produced a soluble oligomer, and accelerated the decline in molecular weight. In the third stage, the soluble oligomer dissolved entirely, and soluble monomer products were obtained [45]. The molecular weight distribution had only two stages: the first stage is 0–60 days, and the second stage is 60 days. The second stage had a lower rate of change than the early stage.

### 3.7. Analysis of Irritation Experiments

**Figure 8:**
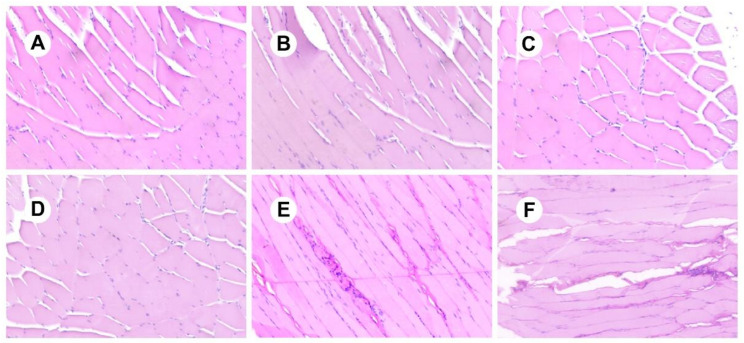
Tissue sections of the implants. (**A**) 0 days; (**B**) 15 days; (**C**) 30 days; (**D**) 60 days; (**E**) 90 days; (**F**) 120 days.

As shown in Figure 8, the RIS-IMs showed no signs of tissue irritation in the body than the control (0 days), indicating that the stimulant had good biocompatibility.

### 3.8. Studying the Stability of RIS-IMs

The RIS-IMs were subject to a six-month stability study under accelerated and long-term conditions. The stability assays were conducted, and the impurities and dissolution of the RIS-IMs were regularly checked. According to the stability data in Table 6, there was almost no change in the quality of the formulation. When the relative humidity was at 40 °C, only a slight decrease in PLA was observed, with no significant release effect. The results showed that the RIS-IMs had good stability.

## 4. Conclusions

This study developed a long-acting RIS-IM that reduces fluctuations in blood drug concentration, decreases the frequency of drug use, and maintains drug release In Vivo. Cumulative release, drug loading, and entrapment efficiency were used as evaluation indicators to evaluate the effects of different pore formers, polymer ratios, porogen concentrations, and oil–water ratios on the RIS-IM. In addition, the correlation coefficient was used as the response value, and a mathematical model was developed by stepwise regression to identify the optimized formulation. The optimized formulation of RIS microspheres had an appropriate particle size, high drug loading, in-vitro release with zero-order kinetics, stable release for more than three months In Vivo, and good stability. In summary, the RIS-IM has obvious advantages and sound potential for clinical application for a broad range of purposes, such as treating schizophrenia.

## Figures and Tables

**Table 1:** Formulation of microspheres.

Formulation	Oil Phase			Inner Water Phase		Internal Water Phase/Oil Phase (*w*/*o*) **
	RIS (g)	PLA (g)	DCM (mL)	Types of Salt	Concentration (%)	
F1	5	5	30	Sucrose	10	1:10
F2	5	5	30	Sodium chloride	10	1:10
F3	7.5	7.5	25	Sodium chloride	5	1:10
F4	6.0	9.0	25	Sodium chloride	5	1:10
F5	7.5	7.5	25	Sodium chloride	20	1:10
F6 *	7.5	7.5	25	-	0	0

* F6 was prepared by the single emulsion method. ** Internal water phase/oil phase is volume/volume ratio.

**Table 2:** Independent variable correlation test results.

	Risperidone/PLA	DCM	PLA Concentration	Sodium Chloride Concentration	*w*/*o*	R^2^
Risperidone/PLA	1.000	0.333	−1.000	0.192	−0.293	0.205
DCM	0.333	1.000	−0.333	−0.192	0.410	−0.381
PLA concentration	−1.000	−0.333	1.000	−0.192	0.293	−0.205
Sodium chloride concentration	0.192	−0.192	−0.192	1.000	0.542	−0.636
*w*/*o*	−0.293	0.410	0.293	0.542	1.000	−0.992
R^2^	0.205	−0.381	−0.205	−0.636	−0.992	1.000

**Table 3:** Diagnosis of the regression model.

	Value	*p*-Value	Decision
Global Stat	1.366	0.850	Assumptions acceptable.
Skewness	0.202	0.653	Assumptions acceptable.
Kurtosis	0.159	0.690	Assumptions acceptable.
Link Function	0.208	0.648	Assumptions acceptable.
Heteroscedasticity	0.797	0.372	Assumptions acceptable.

**Table 4:** Physical and chemical properties of different microsphere formulations.

Formulation	Particle Size (µm)	Specific Surface Area (m^2^/kg)	Drug Loading of Microsphere (%)	Drug Loading of the Implant (%)	Entrapment Efficiency of Implant (%)	Correlation Coefficient (R^2^)
F1	133.7 ± 5.2	65.44	25.5 ± 3.2	32.9 ± 2.0	65.8 ± 4.0	0.918
F2	138.9 ± 6.2	38.24	34.6 ± 3.1	41.0 ± 3.1	82.0 ± 6.2	0.860
F3	125.5 ± 5.2	29.39	38.1 ± 3.5	42.2 ± 3.1	84.4 ± 6.2	0.875
F4	139.0 ± 6.8	28.88	31.1 ± 3.5	36.9 ± 1.6	92.3 ± 4.0	0.977
F5	166.7 ± 8.7	21.68	29.3 ± 5.8	37.3 ± 3.1	74.6 ± 1.6	0.858
F6	135.4 ± 4.5	29.77	40.0 ± 4.3	45.3 ± 2.7	90.6 ± 5.4	0.918
OP	134.4 ± 3.5	30.77	42.1 ± 4.2	46.7 ± 2.3	93.4 ± 4.6	0.997

R^2^: by least squares method.

**Table 5:** Pharmacokinetic parameters of implants.

Parameter	Unit	Dose	
		25 mg	50 mg
t_1/2_	d	19.00	18.40
T_max_	d	75.00	16.00
C_max_	ng/mL	49.07	77.22
AUC_0-t_	ng/mL·d	4197	6594
AUC 0-inf_obs	ng/mL·d	4274	6685
AUC 0-t/0-inf_obs		0.9820	0.9864
MRT 0-inf_obs	d	69.57	60.68
Vz/F_obs	(mg)/(ng/mL)	0.1603	0.1981
Cl/F_obs	(mg)/(ng/mL)/d	5.800 × 10^−3^	7.5 × 10^−3^

**Table 6:** Investigation data of implant stability.

	Packaging Material	Storage Conditions	Time	Experimental Project	Results
Accelerated test	Brown 10-mL vial rubber stopper	40 °C 60% RH	6 months	Assay, Impurities, Dissolution	The Mw of PLA decreased from 48,000 to 43,500, and the drug release and impurities of the implants did not change significantly.
Long-term test	Brown 10-mL vial rubber stopper	5 °C	6 months	Assay, Impurities, Dissolution	There was no significant change in the Mw of PLA, drug release, and impurities.

## Data Availability

The data presented in this study are available on request from the corresponding author. The data are not publicly available due to business purposes.
